# Radial peripapillary capillary density in superior segmental optic hypoplasia measured with OCT angiography

**DOI:** 10.1186/s12886-020-01453-6

**Published:** 2020-05-24

**Authors:** Maiko Abe, Kazuko Omodaka, Tsutomu Kikawa, Toru Nakazawa

**Affiliations:** 1 Department of Ophthalmology, Tohoku University Graduate School of Medicine, Sendai, Japan; 2 Department of Ophthalmic Imaging and Information Analytics, Tohoku University Graduate School of Medicine, Sendai, Japan; 3 R&D Division, Topcon Corporation, Tokyo, Japan

**Keywords:** Optical coherence tomography angiography, Diagnosis, Radial peripapillary capillary density, Superior segmental optic hypoplasia

## Abstract

**Background:**

To investigate the diagnostic power of radial peripapillary capillary (RPC) density, measured with optical coherence tomography angiography (OCT-A), in patients with superior segmental optic hypoplasia (SSOH).

**Methods:**

Forty subjects with SSOH and 40 age- and axial length-matched control subjects were retrospectively registered for this study. SSOH was defined as intraocular pressure less than 21 mmHg with the presence of two of the following: superior rim thinning, superior entrance of the central retinal artery, scleral halo, and pale optic disc; as well as non-progressive visual field loss. RPC density was measured with swept-source OCT-A (Triton, Topcon) overall, in the quadrants, and in the 12 clock-wise sectors. Changes in RPC density were also compared in SSOH patients and age-matched patients with mild- or moderate-stage of glaucoma. RPC density was compared in pairs of groups with Welch’s t-test. Diagnostic power was assessed with the area under the receiver operating characteristics curve (AUC).

**Results:**

Overall cpRNFLT was significantly different in the normal (106.7 ± 9.5 μm) and SSOH (77.2 ± 13.7 μm, *p* <  0.001) subjects. RPC density overall and in the superior, nasal, and inferior quadrants was significantly lower in the SSOH group (all, *p* <  0.001), but not in the temporal (*p* = 0.756) quadrant. The diagnostic power of RPC density was highest in the superior quadrant (AUC = 0.928) and the 1 o’clock sector (0.896). Comparing the SSOH and glaucoma patients showed that there were no significant differences in RPC density either overall (*p* = 0.391) or in the superior quadrant (*p* = 0.268), while RPC density was significantly higher in the inferior (*p* = 0.005) and temporal quadrants (*p* <  0.001) and lower in the nasal quadrant (*p* = 0.029).

**Conclusions:**

Low RPC density was found in the three non-temporal quadrants of the optic nerve head in SSOH patients, in comparison to normal subjects. Regionally, RPC density in SSOH was lower in the nasal quadrant and higher in the inferior and temporal quadrants in comparison to glaucoma patients. Measuring RPC density with OCT-A may help the diagnosis of SSOH and may improve the management of glaucoma.

## Background

Superior segmental optic hypoplasia (SSOH) is a congenital anomaly of the optic nerve head (ONH) characterized by superior entry of the central retinal vessels, superior peripapillary scleral halo, and reduced circumpapillary retinal nerve fiber layer thickness (cpRNFLT) with non-progressive inferior-sector visual field defects. In Asia, normal-tension glaucoma (NTG) is the primary subtype of open-angle glaucoma [1] making it important to differentiate SSOH from NTG [2]. Previously, it was reported that SSOH could be identified based on the location of cpRNFLT loss [3] and structural abnormalities in the extension of the retinal pigment epithelium (RPE) over the nasal disc margin [4], both measured with spectral-domain optical coherence tomography (OCT). Thus, both fundus photography and OCT may be useful in the clinical treatment of SSOH.

We previously showed that tissue mean blur rate (MT), representing blood flow (BF) in the ONH, decreases in SSOH [5]. MT is also correlated with deep-ONH BF, but not surface-ONH BF [6]. Recently, OCT angiography (OCT-A), based on swept-source OCT, has become available clinically and is increasingly important for visualizing vessels both in the retina and ONH. The density of radial peripapillary capillaries (RPCs), a network of surface nerve fiber layers, has been shown to have diagnostic power for glaucoma [7]. However, until now, there have been no reports on changes in RPC density or the diagnostic power of RPC density in SSOH. This study is thus the first to assess the microvasculature of the peripapillary retina and investigate whether vascular parameters are useful for diagnosing SSOH.

## Methods

Eighty eyes of 40 normal and 40 SSOH patients were included. SSOH was diagnosed by glaucoma specialists after a careful differential diagnosis for glaucoma [4]. SSOH diagnosis was based on the presence of more than two of the following four symptoms: superior rim thinning, superior entrance of the central retinal artery, scleral halo, and pale optic disc; combined with non-progressive visual field loss (in an average of 6.3 ± 4.8 visual field tests), peripheral visual field defects in the V-4 target of the Goldmann visual field test, and intraocular pressure (IOP) less than 21 mmHg. Mean deviation measurements used reliable data from Humphrey field analyzer standard automated perimetry. We also recruited 50 separate patients, including 27 SSOH patients and 23 age-matched mild- or moderate-stage glaucoma patients, to compare regional differences in RPC density. RPC density was measured with OCT-A (Triton, Topcon), as previously described [7], overall, in the superior, temporal, inferior, and nasal quadrants, and in 12 clock-wise sectors. Swept-source OCT (SS-OCT) uses a tissue-penetrating laser system with a long central wavelength of 1050 nm and allows patients to easily maintain good fixation during scanning due to its low glare. The device is also equipped with an eye-tracking system. Thus, OCT-A with SS-OCT has led to significant improvements in the observation of retinal capillaries, both at the surface and in the deep areas of the ONH, in glaucoma patients. cpRNFLT was measured with swept-source OCT.

This study adhered to the tenets of the Declaration of Helsinki, and the protocols were approved by the institutional review board of the Tohoku University Graduate School of Medicine (study 2017–1-290). Clinical findings and other characteristics, including age, sex, axial length, and IOP, were compared in pairs of groups with Welch’s t-test. The area under the receiver operating characteristic curve (AUC) was calculated for differentiating normal subjects and SSOH patients based on OCT-A parameters. All statistical analyses were performed with JMP software (Pro version 13.1.0, SAS Institute Japan Inc., Tokyo, Japan).

## Results

**Fig. 1:**
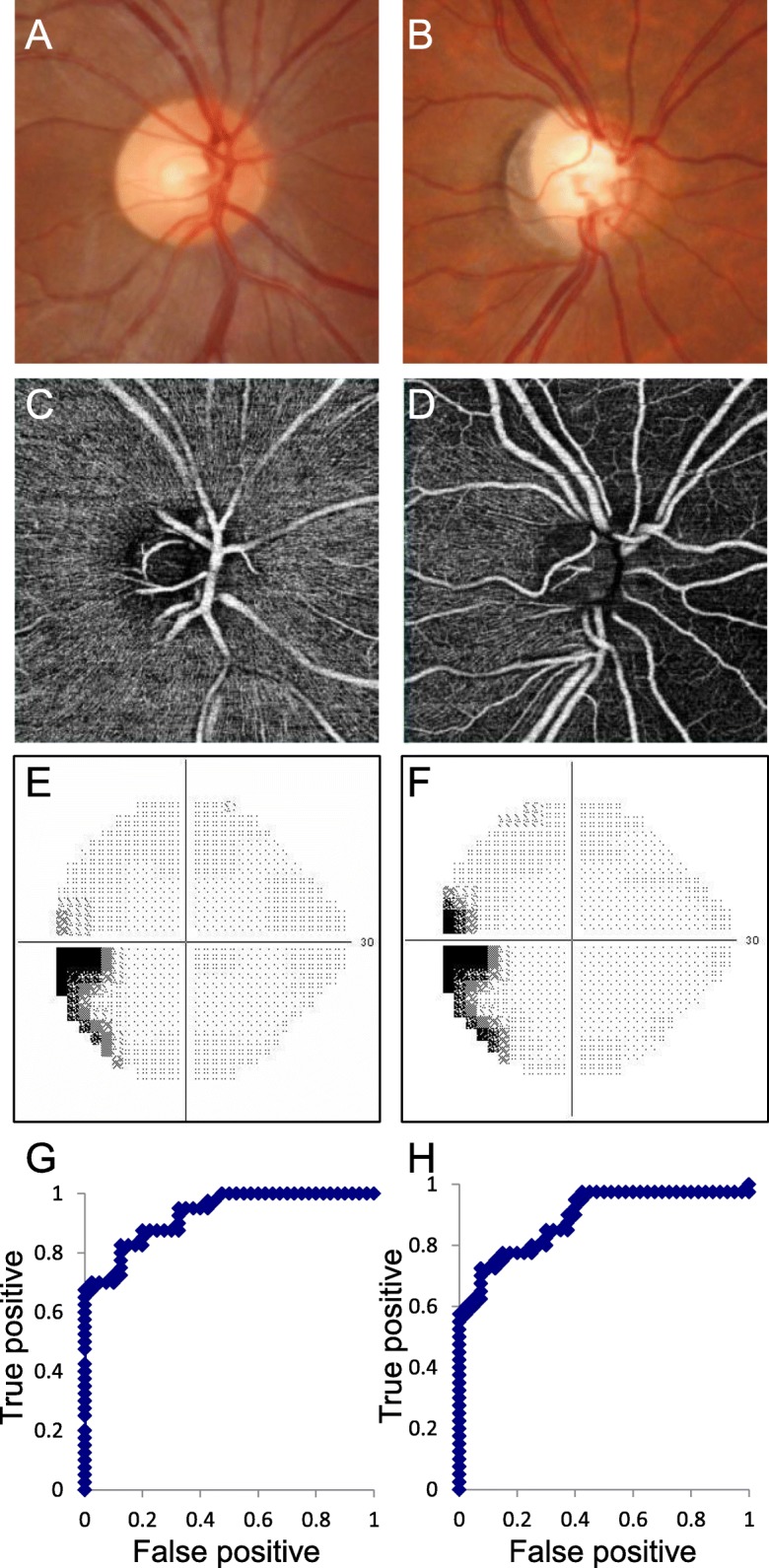
Representative normal and SSOH subjects and the regional diagnostic power of RPC density for SSOH. **a, b**: Fundus photography. **c, d**: RPC density measured with OCT-A. A, C: Normal subject. **b, d**: SSOH subject. **e, f**: Grayscale map of the visual field obtained with the Humphrey field analyzer in the SSOH patient (E: baseline, F 3 years later). G Receiver operating characteristic (ROC) curve for superior-quadrant cpRNFLT. H: ROC curve for the 1 o’clock sector of cpRNFLT

There were no differences between the groups in age (normal: 39.5 ± 8.2, SSOH: 41.2 ± 18.0, *p* = 0.326), axial length (24.6 ± 0.8, 25.1 ± 1.3, *p* = 0.088), or IOP (14.0 ± 2.2, 14.1 ± 2.5, *p* = 0.749). Figure 1 shows a representative SSOH patient without visual field progression over 5 years and a normal subject; RPC density in the superior to nasal quadrants is clearly lower in the SSOH subject. Table 1 shows differences in RPC density between the normal and SSOH groups. There were significant differences in overall RPC density and in the superior, nasal, and inferior (all: *p* <  0.001), but not temporal (*p* = 0.756) quadrants. RPC density was significantly lower in the 1, 2, 4, 5, 7, 9, 11, and 12 o’clock sectors. Table 2 shows the diagnostic power of RPC density. The highest AUC was in the superior quadrant (AUC = 0.928, Fig. 1e), with a cutoff value of 42.14% (83% sensitivity and 88% specificity). The 1 o’clock sector had the highest AUC (0.896, Fig. 1f), with a cutoff value of 34.8% (80% sensitivity and 83% specificity).

**Table 1:** Comparison of RPC density in normal and SSOH subjects

	RPC (%)		
	Normal (n = 40)	SSOH (*n* = 40)	*p*
Average	46.1 ± 3.3	39.6 ± 4.7	< 0.001***
Superior	50.3 ± 7.1	36.6 ± 7.1	< 0.001***
Nasal	37.6 ± 5.7	31.5 ± 8.1	< 0.001***
Inferior	50.7 ± 5.8	44.0 ± 10.2	< 0.001***
Temporal	45.9 ± 5.6	46.3 ± 6.6	0.756
Superior			
11	56.6 ± 8.4	49.6 ± 10.7	0.002**
12	43.0 ± 11.7	30.5 ± 13.3	< 0.001***
1	51.5 ± 11.2	30.3 ± 12.9	< 0.001***
Nasal			
2	42.7 ± 9.5	33.6 ± 13.7	< 0.001***
3	31.6 ± 8.1	29.0 ± 10.9	0.238
4	38.7 ± 7.8	32.0 ± 11.7	0.004**
Inferior			
5	45.9 ± 9.9	36.8 ± 12.5	< 0.001***
6	44.9 ± 8.5	41.3 ± 13.6	0.158
7	61.3 ± 9.0	54.9 ± 11.3	0.007**
Temporal			
8	46.5 ± 8.3	47.2 ± 8.6	0.700
9	38.2 ± 7.3	41.9 ± 8.1	0.039*
10	52.7 ± 8.9	50.0 ± 10.7	0.226

**P <  0.01, ***P < 0.001

**Table 2:** Diagnosis power of localized RPC density in normal and SSOH subjects

	AUC of RPC (%)
Average	0.879
Superior	0.928
Nasal	0.744
Inferior	0.706
Temporal	0.520
Superior	
11	0.692
12	0.769
1	0.896
Nasal	
2	0.729
3	0.637
4	0.693
Inferior	
5	0.709
6	0.614
7	0.669
Temporal	
8	0.513
9	0.628
10	0.563

We also compared changes in RPC density in 27 SSOH patients and 23 age-matched patients with mild- or moderate-stage of glaucoma. There were no differences between the groups in age (normal: 39.5 ± 8.2, SSOH: 41.2 ± 18.0, *p* = 0.326), IOP (14.0 ± 2.2, 14.1 ± 2.5, *p* = 0.749) or axial length (24.6 ± 0.8, 25.1 ± 1.3, *p* = 0.088), but there was a significant difference in mean deviation (24.6 ± 0.8, 25.1 ± 1.3, *p* = 0.088). We found that RPC density in the SSOH patients was significantly higher in the inferior (*p* = 0.005, 0.725) and temporal (*p* <  0.001, 0.781) quadrants and lower in the nasal quadrant (*p* = 0.029, 0.688). There was no difference in overall RPC density (*p* = 0.391, AUC = 0.568) or RPC density in the superior quadrant (*p* = 0.268, 0.593), as shown in Table 3.

**Table 3:** Comparison of RPC density in SSOH and glaucoma subjects and the diagnostic power of regional RPC density for SSOH

	RPC (%)			AUC
	SSOH (*n* = 27)	Glaucoma (*n* = 23)	*P* value	
Average	57.3 ± 11.1	54.7 ± 10.4	0.391	0.568
Superior	56.8 ± 15.7	62.3 ± 18.7	0.268	0.592
Nasal	35.7 ± 19.1	46.8 ± 15.4	0.029*	0.688
Inferior	68.5 ± 15.8	55.8 ± 14.9	0.005**	0.725
Temporal	68.7 ± 11.0	55.0 ± 13.0	< 0.001***	0.781

*P < 0.05, **P < 0.01, ***P < 0.001

## Discussion

We found that the power of RPC density to differentiate SSOH patients and controls was strong, especially in the superior, nasal, and inferior areas. Previously, we used laser speckle flowgraphy to show that SSOH, a congenital anomaly, causes reduced microcirculation in the ONH [5], that the quadrant MT ratios in the ONH have a strong power to differentiate SSOH and NTG (indicating that SSOH has a fundamentally different pathogenesis from NTG) [5], and that the structure of the deep layers of the ONH, around the lamina cribrosa, are correlated to MT, but not RPC density [6]. Here, we found that the density of the peripapillary retinal microvasculature decreased in SSOH, but that the pattern of RPC loss was distinctly different from that reported for cpRNFLT [3]. RPC density in the 9 o’clock sector, outside the temporal area, was significantly lower, but RPC density in the 3 o’clock sector was not significantly different in the SSOH and normal subjects. On the other hand, cpRNFLT was not significantly different in the 9 o’clock sector (normal: 67.6 ± 9.9, SSOH: 70.3 ± 19.4, *p* = 0.431), but was in the 3 o’clock sector (normal: 63.6 ± 12.7, SSOH: 57.8 ± 26.9, *p* <  0.001). Thus, the location of changes in RPC density and cpRNFLT is on horizontally opposite sides of the ONH. This may be due to lower initial RPC density in the nasal ONH, higher RPC density in the temporal ONH, and the location of the major retinal central vessel in the superior and inferior ONH. These findings suggest that OCT-A might have the potential to diagnose SSOH.

In this study, we also performed an investigation to compare SSOH patients and age-matched mild- and moderate-stage glaucoma patients. RPC density in the SSOH patients was not significantly different overall or in the superior quadrant but was significantly higher in the inferior and temporal quadrants and lower in the nasal quadrant. These differences in RPC density are understandable, because the area of the nerve fiber layer that is vulnerable to damage is different in SSOH and glaucoma. Our finding also shows that RPC density may be valuable for assessing damage in SSOH. Theoretically, cpRNFLT damage in SSOH is developmental, while damage in glaucoma is secondary. However, the diagnostic power of RPC density to differentiate SSOH and glaucoma was not strong, and further study is needed to determine the relationship between cpRNFLT and vasculature.

Limitations of this cross-sectional study include a small size. This study is the first to investigate changes in RPC density in SSOH. Generally, sample size calculations are hard to perform in such exploratory studies. Another limitation was the use of a specific manufacturer-dependent method for the evaluation of RPC density, even though there are currently several differing methods of calculating RPC density with devices from different companies. We excluded patients with high myopia and applied a method [8] to compensate for optical magnification of the eye, and excluded the major central retinal vessels by using image processing with a Laplacian of Gaussian filter with noise reduction. Thus, we are the first to demonstrate the potential of RPC density measurement for SSOH diagnosis.

## Conclusion

We found that RPC density decreased in SSOH, and that changes in microcirculation were indicative of SSOH. However, it remains unclear whether these changes are congenital or secondary to retinal nerve fiber layer degeneration. In addition to cpRNFLT and the extension of the RPE over the nasal disc margin [4], OCT-A may be a new, non-invasive, objective instrument for SSOH diagnosis.

## Data Availability

The datasets used and/or analysed during the current study are available from the corresponding author on reasonable request.

## Abbreviations

AUC: Area under the receiver operating characteristic curve
BF: Blood flow
cpRNFLT: Circumpapillary retinal nerve fiber layer thickness
IOP: Intraocular pressure
MT: Tissue-area mean blur rate
OCT-A: Optical coherence tomography angiography
ONH: Optic nerve head
RPC: Radial peripapillary capillary
RPE: Retinal pigment epithelium
SSOH: Superior segmental optic hypoplasia
